# Detection of Influenza Virus Infection Using Two PCR Methods

**DOI:** 10.1155/2014/274679

**Published:** 2014-12-09

**Authors:** Richard K. Zimmerman, Charles R. Rinaldo, Mary Patricia Nowalk, G. K. Balasubramani, Mark G. Thompson, Arlene Bullotta, Michael Susick, Stephen Wisniewski

**Affiliations:** ^1^Department of Family Medicine, University of Pittsburgh School of Medicine, 3518 5th Avenue, Pittsburgh, PA 15261, USA; ^2^Department of Pathology, University of Pittsburgh School of Medicine, PA 15261, USA; ^3^Department of Infectious Disease and Microbiology, University of Pittsburgh Graduate School of Public Health, Pittsburgh, PA 15261, USA; ^4^Department of Epidemiology, University of Pittsburgh Graduate School of Public Health, Pittsburgh, PA 15261, USA; ^5^Influenza Division, Centers for Disease Control and Prevention, Atlanta, GA 30307, USA

## Abstract

Rapid, accurate, and cost-effective methods to identify the cause of respiratory tract infections are needed to maximize clinical benefit. Outpatients with acute respiratory illness were tested for influenza using a singleplex reverse transcriptase polymerase chain reaction (SRT-PCR) method. A multiplex RT-PCR (MRT-PCR) method tested for influenza and 17 other viruses and was compared with SRT-PCR using chi-square tests. Among 935 patients, 335 (36%) tested positive for influenza A and influenza B using SRT-PCR. Using MRT-PCR, 320 (34.2%) tested positive for influenza A and influenza B. This study supports MRT-PCR as a comparable method for detecting influenza among patients seeking outpatient care for acute respiratory illnesses.

## 1. Background

Each year millions of people are afflicted with influenza-associated respiratory tract infections, some of which lead patients to seek outpatient medical attention, referred to as medically attended acute respiratory infection (ARI). Rapid, accurate, and cost-effective methods are needed to maximize clinical benefit of antivirals and reduce inappropriate antibiotic usage. New assay methods using multiplex reverse transcriptase polymerase chain reaction (MRT-PCR) are available that allow for relatively rapid detection of multiple virus types including influenza [1, 2].

## 2. Objective

During the 2012-2013 influenza season, the Centers for Disease Control and Prevention (CDC)-funded, multicenter US Influenza Vaccine Effectiveness (Flu VE) Network conducted a study designed to determine the effectiveness of the season's influenza vaccine. That study used singleplex RT-PCR (SRT-PCR) to detect influenza virus. The University of Pittsburgh site of the Flu VE Network also used MRT-PCR. The purpose of this study was to compare the agreement between SRT-PCR and MRT-PCR for influenza virus detection.

## 3. Study Design

The US Flu VE Network evaluated the effectiveness of the season's influenza vaccine using a test-negative, case-control study design [3, 4], where the proportion vaccinated among those who test positive for influenza was compared with the proportion vaccinated among those who test negative. Participants in the current study were enrollees in the University of Pittsburgh site of the Flu VE study, described previously [5]. Eligibility criteria included patients aged ≥6 months as of 9/1/2012, seeking outpatient medical care for an upper respiratory illness of ≤7 days' duration with cough, and not taking an influenza antiviral before the visit. This prospective study was approved by the University of Pittsburgh Institutional Review Board.

Nasal and oropharyngeal mucosa were each sampled using polyester swabs that were combined in the same cryovial and delivered to the UPMC Clinical Virology Laboratory within 72 hours. The specimens were stored in a lysis buffer and aliquoted for nucleic acid isolation and detection of influenza virus using CDC's singleplex RT-PCR (SRT-PCR) test and a MRT-PCR test using the eSensor XT-8 instrument and respiratory viral panel from GenMark Diagnostics, Inc.* In toto*, 1171 specimens were collected and tested for presence of influenza using SRT-PCR. Of those, funding was available to analyze with MRT-PCR all specimens SRT-PCR-positive for influenza and a random sample of specimens SRT-PCR-negative for influenza, for a total of 935 specimens that were doubly assayed. The purpose of this study was to compare the agreement between SRT-PCR and MRT-PCR for influenza virus detection.

Both the SRT-PCR and MRT-PCR extraction and assay methods have been previously published [1, 6, 7]. The eSensor RVP MRT-PCR assay (GenMark Diagnostics) used in this study is currently approved for clinical use in Europe. It has the same methodological characteristics but a broader range of viral analytes than the US FDA-cleared version and includes seasonal influenza A virus (H1N1 and H3N2 subtypes) and influenza B virus.

The measurement of agreement between the SRT-PCR and MRT-PCR influenza assays was determined using Cohen's kappa statistic. Analyses were conducted using SAS version 9.2 (SAS Institute, Inc., Cary, NC).

## 4. Results

Among 935 ARI patients, influenza A virus was detected in 260 specimens and influenza B virus was detected in 76 specimens using SRT-PCR, and influenza A virus was detected in 251 specimens and influenza B virus was detected in 69 specimens using MRT-PCR. There was significant agreement on influenza results between assays; for influenza A, kappa = 0.97, 95% confidence intervals (95% CI) = 0.95–0.99, and Chi-square *P* < 0.001; for influenza B, kappa = 0.95, 95% CI = 0.91–0.99, and Chi-square *P* < 0.001; and for overall results, kappa = 0.96, 95% CI = 0.94–0.98, and Chi-square *P* < 0.001. The sensitivity was 96% and 91% and specificity was 99.8% and 100%, respectively, for influenza A and influenza B, when SRT-PCR was treated as the gold standard (Table 1).

The mean cycle threshold (Ct) value from SRT-PCR for influenza A was 27.4 (standard deviation (*sd*⁡) = 5, *n* = 250) and for influenza B was 27.2 (*sd*⁡ = 5.4, *n* = 69). Among the SRT-PCR positives but MRT-PCR negatives for influenza A, the Ct values ranged from 21.7 to 39.3, with most (8 of 10 where data were available) being above 32.4 (which was 1 sd above the influenza A mean), suggesting that these were weak positives. Two of these SRT-PCR influenza A positives but MRT-PCR negatives were also positive for another virus in the multiplex assay (respiratory syncytial virus and human rhinovirus). Among the SRT-PCR positives but MRT-PCR negatives for influenza B, the Ct values ranged from 22.2 to 38.7, with most (4 of 7 where data were available) being above 32.6 (which was 1 sd above the influenza B mean), suggesting that these were weak positives. Three of these SRT-PCR influenza B positives but MRT-PCR negatives were also positive for another virus in the multiplex assay (respiratory syncytial virus and coronavirus). Forty-seven percent of the discordant results and 49% of the concordant results were from persons who were vaccinated.

The mean Ct value for ribonuclease P (RNaseP, a protein that may reflect sample quality) for 594 persons who were PCR negative was 28 (*sd*⁡ = 2.5) versus 29.1 (*sd*⁡ = 2.8) for those who were PCR positive. The one person who was MRT-PCR positive for influenza A but SRT-PCR negative had a RNaseP value of 24.7, suggesting that the sample quality was good. The RNaseP values for the SRT-PCR positive but MRT-PCR negative ranged from 24.7 to 32.9, with 3 specimens being higher than 31.9 (a level >1 sd above the RNaseP values for the other positives), suggesting that sample quality was good in the majority of such specimens.

## 5. Discussion

For influenza, the multiplex corresponded well with singleplex RT-PCR with high kappa values. In a previous study examining concordance between SRT-PCR and MRT-PCR, Zimmerman et al. [7] reported a kappa of 0.83 (95% CI = 0.75–0.92) for 2011-2012 when only 3 of the influenza strains identified were influenza B. Some of the discordant samples were positive for another virus in MRT-PCR or weak positives by RT-PCR. The sensitivity (96% for influenza A and 91% for influenza B) and specificity (100% for influenza A and 100% for influenza B) were higher in the present study than previously reported (overall sensitivity = 91.1% and specificity = 98.2%) [7]. This might be due to differences in influenza viruses (i.e., a late variation in the predominant H3N2 in 2011-12 season was noted) [8].

### 5.1. Strengths and Limitations

This study offers the ability to compare influenza detection using two assays during a winter influenza season in which both A and B strains were circulating. We were unable to test an inclusive sample of all of the ARI specimens with the MRT-PCR. However, the sample size and strength of the sensitivity and specificity analysis were sufficient to allow confidence in the use of the MRT-PCR for detecting both influenza A and B viruses.

## 6. Conclusions

This study supports the similarity between SRT-PCR and MRT-PCR for detecting influenza virus among patients seeking outpatient care for acute respiratory illnesses.

## Figures and Tables

**Table 1:** Comparison of singleplex reverse transcriptase polymerase chain reaction (SRT-PCT) with multiplex reverse transcriptase polymerase chain reaction (MRT-PCR) showing sensitivity and specificity of MRT-PCR compared to SRT-PCR for influenza, 2012-2013.

Influenza A				Influenza B			
MRT-PCR	SRT-PCR			MRT-PCR	SRT-PCR		
	Positive	Negative	Total		Positive	Negative	Total
Positive	250	1	251	Positive	69	0	69
Negative	10	672	682	Negative	7	851	858
Total	**260**	**673**	**933** ^*^	Total	**76**	**851**	**927** ^*^
Sensitivity/specificity	96%	99.8%		Sensitivity/specificity	91%	100%	

MRT-PCR: multiplex reverse transcriptase polymerase chain reaction; SRT-PCR: singleplex reverse transcriptase polymerase chain reaction.

^*^Of the 935 specimens, 2 specimens in SRT-PCR had inconclusive results/missing data (leaving 933) and were MRT-PCR negative for influenza A and 8 specimens in SRT-PCR had inconclusive results/missing data (leaving 927) and were MRT-PCR negative for influenza B.
